# MicroRNA-146b-3p regulates the dysfunction of vascular smooth muscle cells via repressing phosphoinositide-3 kinase catalytic subunit gamma

**DOI:** 10.1080/21655979.2021.1937904

**Published:** 2021-06-11

**Authors:** Xijing Zhuang, Feng Gao, Lei Shi, Wei Liu, Wenjun Wang, Xuezhi He, Yang Gao

**Affiliations:** Department of Cardiac Surgery, Dalian Municipal Center Hospital, Dalian Liaoning, China

**Keywords:** Mir-146b-3p, vascular smooth muscle cells, phosphoinositide-3 kinase catalytic subunit-gamma, platelet-derived growth factor-bb

## Abstract

MicroRNAs are crucial regulators in the phenotype switch of vascular smooth muscle cells (VSMCs). Nonetheless, the role of miR-146b-3p in VSMCs remains unclear. In the present study, platelet-derived growth factor-BB (PDGF-BB) at different concentrations was employed to stimulate VSMCs for different times, to establish the model of VSMC dysfunction. The relative expression of miR-146b-3p was quantified by quantitative real-time polymerase chain reaction (qRT-PCR). The proliferation of VSMCs was measured by BrdU assay. Flow cytometry analysis was employed for the analysis of cell cycle. VSMC migration was detected by Transwell assay. Phosphoinositide-3 kinase catalytic subunit-gamma (PIK3CG) and markers of VSMC differentiation, including α-SMA, SM-22α, SMMHC, and Calponin were examined employing Western blot. The targeting relationship between miR-146b-3p and PIK3CG 3ʹ-UTR was affirmed by dual-luciferase gene assay. We report that the reduction of miR-146b-3p expression was induced by PDGF-BB in a time-dependent and dose-dependent manner (*P* < 0.05). The overexpression of miR-146b-3p counteracted the effects of PDGF-BB on the proliferation and migration of VSMCs and increased the expressions of differentiation markers (*P* < 0.05). Additionally, PIK3CG expression was negatively regulated by miR-146b-3p, and the restoration of PIK3CG partly eliminated the effects of miR-146b-3p on VSMCs (*P* < 0.05). In summary, miR-146b-3p represses the proliferation, migration, and phenotype switch of VSMCs induced by PDGF-BB via targeting PIK3CG. Therefore, miR-146b-3p/PIK3CG may be a potential target for the treatment of atherosclerosis.

## Graphical Abstract

**Figure:**
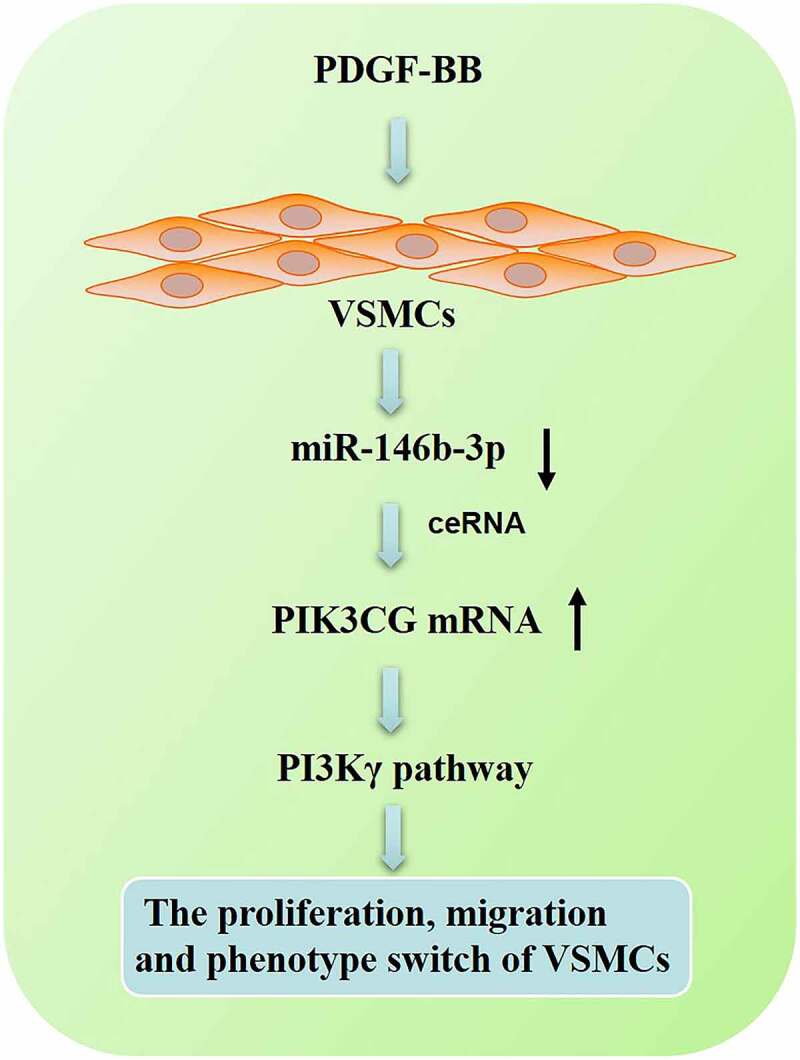

## 1 Introduction

Vascular smooth muscle cells (VSMCs) are the main components in the media of blood vessels, and the maintenance of VSMC’s contractile phenotype is crucial to the normal structure and the function of blood vessels [1]. Stimuli such as excessive lipids trigger phenotypic alteration of VSMCs, from contractile phenotype to synthetic phenotype, facilitating the proliferation and migration of VSMCs, further improving the secretion of extracellular matrix, and ultimately promoting the pathogenesis of atherosclerosis (AS) [2,3]. The dysfunction of VSMCs also contributes to the formation and destabilization of AS plaque and mediates the inflammatory response [4]. The migration and proliferation of VSMCs are regulated by many factors, including platelet-derived growth factor-BB (PDGF-BB) [5]. Therefore, exploring the potential mechanism of PDGF-BB-mediated VSMCs proliferation and migration is valuable to find effective targets to prevent or reverse the occurrence and development of AS.

MicroRNAs (miRNAs) participate in the phenotypic transformation and dysfunction of VSMCs [6]. For instance, after the treatment of VSMCs with PDGF-BB, miR-124 expression is remarkably down-regulated in VSMCs; the augmentation of miR-124 expression induces the up-regulation of expressions of VSMC differentiation markers, such as smooth muscle actin (SMA) and smooth muscle 22 alpha (SM22α), and reduces the proliferation and migration of VSMCs [7]. Previous studies report the functions of miR-146b-3p in cancer. For example, miR-146b-3p expression is up-regulated in cervical cancer tissues compared with that in adjacent tissues and miR-146b-3p promotes colony formation and migration of cancer cells [8]. Besides, miR-146b-3p can repress the proliferation and differentiation of chicken myoblasts and expedite its apoptosis by inhibiting PI3K/AKT pathway [9]. At present, little is known about the exact function of miR-146b-3p in regulating the phenotypes of VSMCs,

AS one of the catalytic subunits of phosphoinositide-3 kinase (PI3K), PI3Kγ is encoded by phosphoinositide-3 kinase catalytic subunit gamma (PIK3CG) gene [10]. In a cohort study enrolling 3669 patients, reportedly, the mutation of PIK3CG is significantly correlated with the formation of AS plaque and carotid intima-media thickness [11], indicating an association between PI3Kγ/PIK3CG and the development of AS. Moreover, knockdown of PIK3γ leads to a remarkable enhancement of expressions of differentiation markers Calponin and SM22α in fetal bovine serum (FBS)-treated VSMCs; the arterial intimal hyperplasia in PIK3γ knockout mice is ameliorated [12].

Our bioinformatics analysis implied that miR-146b-3p was a potential modulator in AS development, and PIK3CG was a candidate target of miR-146b-3p. So we hypothesized that miR-146b-3p could probably modulate the dysfunction of VSMCs. In this research, PDGF-BB was used to induce the proliferation and migration of VSMCs, and the impacts of miR-146b-3p on the VSMCs were investigated. It was demonstrated that miR-146b-3p inhibited proliferation and migration and reversed the phenotype switch of VSMCs via targeting PIK3CG. These findings provided a new scientific basis and new ideas for the treatment of AS.

## 2 Materials and methods

### 2.1 Microarray analysis and cell culture

Gene Expression Omnibus database (GEO) (http://www.ncbi.nlm.nih.gov/geo) is a public genomic database. Four datasets of microarray data [Gene Expression Series (GSE)127,016, GSE26555, GSE28829, GSE38574] were downloaded from GEO [13–15]. In GSE127016, the transcriptional profiles of VSMCs treated with TNF-α and control VSMCs are compared. In GSE26555, the miRNA expression profile in AS plaques induced by disturbed blood flow was analyzed. In GSE28829, the genes in human AS plaques at early stage and advanced stage are compared. In GSE38574, gene expression profiles of the tissues from the aortic arch, of the mice model at different stages of AS development, are compared. These datasets were reanalyzed to screen out the novel regulators of AS development.

Human VSMCs from the American Type Culture Collection (ATCC, Rockville, MD, USA) were cultured in RPMI-1640 medium (Thermo Fisher Scientific, Waltham, MA, USA) supplemented with 100 U/ml penicillin, 100 μg/ml streptomycin (Thermo Fisher Scientific, Waltham, MA, USA), and 10% fetal bovine serum (FBS, Thermo Fisher Scientific, Waltham, MA, USA) in 5% CO_2_ at 37°C. To induce the hyperproliferation, excessive migration, and phenotype switch of VSMCs, the cells were treated with PDGF-BB at different concentrations (0, 10, 20, and 40 ng/ml) or different times (0, 6, 12, and 24 h).

Has-miR-146b-3p mimics (5ʹ-UGCCCUGUGGACUCAGUUCUGG-3ʹ), mimics control (mimic_CTL, 5ʹ-UUCUCCGAACGUGUCACGUTT-3ʹ), PIK3CG overexpression plasmids (pcDNA-PIK3CG), and empty vector (pcDNA) were obtained from GenePharma (Shanghai, China). VSMCs in logarithmic growth phase were inoculated into a 12-well plate (1 × 10^5^ cells/well). According to the manufacturer’s instructions, the plasmids or miRNA mimics were transfected into VSMCs with Lipofectamine^TM^ 3000 (Invitrogen, Carlsbad, CA, USA). 36 h later, quantitative real-time polymerase chain reaction (qRT-PCR) was used to detect the transfection efficiency.

### 2.2 qRT-PCR

The extraction of total RNA of VSMCs was accomplished employing TRIzol reagent (Invitrogen, Carlsbad, CA, USA). In conformity with manufacturer’s protocol, the synthesis of cDNA from 1 g of total RNA was performed employing PrimeScript-RT Kit (Promega, Madison, WI, USA), and then qRT-PCR was performed with SYBR®Premix-Ex-Taq™ kit (Takara, Dalian, China) on ABI7300 system (Applied Biosystems, SanFrancisco, CA, USA). U6 and GAPDH were employed as the reference genes of miR-146b-3p and PIK3CG, respectively. The calculation of the relative expression was accomplished utilizing 2^−ΔΔCt^ method.

### 2.3 Cell proliferation and migration assays

BrdU assay was used to detect cell proliferation. Briefly, VSMCs were inoculated into a 35 mm culture dish containing a coverslip, and then the cells were incubated with BrdU solution (Beyotime, Shanghai, China) for 4 h. Subsequently, the cells were fixed in 4% paraformaldehyde for 10 min. Next, anti-BrdU antibody (Biocompare, South San Francisco, CA, USA) was used to incubate the cells at room temperature for 2 h and then the cells were stained with DAPI staining solution (Beyotime, Shanghai, China). After the cells were washed by PBS, five visual fields were randomly selected under a fluorescence microscope (Olympus, Tokyo, Japan), and the average number of BrdU positive cells was calculated.

In cell cycle analysis, VSMCs were fixed with 80% ethanol overnight and then stained with propidium iodide (PI) solution (50 μg/ml, BD Biosciences, Franklin Lakes, NJ, USA) for 30 min at room temperature. Subsequently, after the cells were washed by PBS, the detection of cell cycle was performed on a flow cytometer (BD Biosciences, Franklin Lakes, NJ, USA).

The migration of VSMCs was assessed with Transwell system (Millipore, Bedford, MA, USA). To be specific, 600 μL of RPMI-1640 medium containing 10% FBS was added in the lower compartment, and 100 μL of serum-free RPMI-1640 medium containing 1 × 10^5^ cells was added in the upper compartment. After the cells were cultured at 37°C for 12 h, the remaining non-migrated cells in the upper chamber were wiped off with cotton swabs. The migrated cells were fixed with 4% paraformaldehyde for 10 min and then stained with 0.5% crystal violet. Finally, five visual fields of each membrane were randomly selected under an inverted microscope and the average number of migrated cells in each group was calculated and recorded.

### 2.4 Western blot

VSMCs in each group were collected and lysed in 100 μL of RIPA lysis buffer (Beyotime, Shanghai, China). Then, the total protein was extracted and the protein concentration was determined by Bradford method. The equivalent amount of protein in each group was separated by SDS-PAGE and then transferred onto PVDF membrane (Millipore, Bedford, MA, USA). Then, the membrane was blocked in 5% skim milk at room temperature for 1 h before being incubated with the primary antibody overnight at 4°C. Next, secondary antibody was added and the membranes were incubated at room temperature for 1 h. Electrochemiluminescence substrate A and B solutions (Millipore, Bedford, MA, USA) and Amersham Imager 600 (GE Healthcare, Chicago, IL, USA) were used to visualize the protein bands. The ImageJ software (NIH, Bethesda, Maryland, USA) was utilized for quantifying the protein bands. The antibodies used in this study included anti-PIK3CG (1:1000, 140,307, Abcam, Cambridge, UK), anti-SM-22α (1:1000, ab14106, Abcam, Cambridge, UK), anti-α-SMA (1:1000, ab184705, Abcam, Cambridge, UK), anti-smooth muscle myosin heavy chain 11 (SMMHC) (1:1000, ab133567, Abcam, Cambridge, UK), anti-Calponin (1:1000, ab227661, Abcam, Cambridge, UK), anti-GAPDH (1:3000, 60,004-1-Ig, Proteintech, Wuhan, China), and the secondary antibody (1:2000, SA00001-2, Proteintech, Wuhan, China).

### 2.5 Dual-luciferase reporter assay

The sequence containing the binding site between PIK3CG 3ʹUTR and miR-146b-3p was amplified and cloned into pmirGLO vector (Promega, Madison, WI, USA), and wild-type (WT) luciferase reporter vector was constructed. After the binding site was mutated, mutant (MUT) luciferase reporter vector was obtained. Next, the reporter vectors and miR-146b-3p mimics or mimics control were co-transfected into VSMCs. After 48 h, dual-luciferase reporter kit (Promega, Madison, WI, USA) was used to detect the luciferase activity in each group according to manufacturer’s protocols, and the luciferase activity of firefly was normalized to that of Renilla.

### 2.6 Statistical analysis

In all the *in vitro* experiments, three parallel samples were set for each experiment for biological replicates and each sample was examined three times for technical replicates. SPSS 23.0 software (SPSS Inc., Chicago, IL, USA) was used for statistical analysis. All measurement data were expressed by mean ± standard deviation (x ± s). The comparison between two groups was achieved utilizing *t*-test, and the comparison among multiple groups was made utilizing one-way ANOVA, with *P* < 0.05 indicating the significant difference.

## 3 Results

This study is performed to clarify the biological function of miR-146b-3p in the dysfunction of VSMCs induced by PDGF-BB. Through a series of *in vitro* experiments, we demonstrate that PDGF-BB represses the expression of miR-146b-3p in VSMCs and miR-146b-3p represses the proliferation and migration and promotes the differentiation of VSMCs via suppressing PIK3CG.

### 3.1 The research of miR-146b-3p via bioinformatics analysis

**Figure 1.**
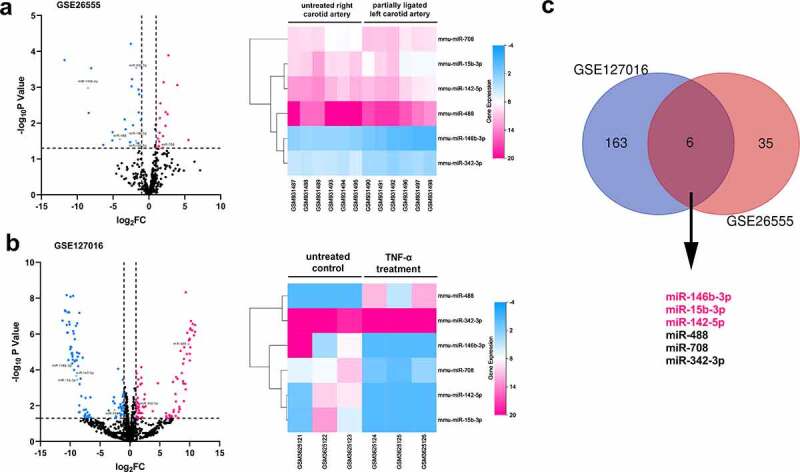
The prediction of pivotal miRNAs in VSMCs which participated in the pathogenesis of AS

In order to explore the pathogenesis of AS, publicly accessible datasets GSE127016 and GSE26555 were analyzed to screen out the miRNAs probably modulated the formation of AS plaque. In the intersection, there are six differentially expressed miRNAs (miR-146b-3p, miR-142-5p, miR-15b-3p, miR-708, miR-342-3p, and miR-488) shared by the two datasets; only miR-146-3p, miR-15b-3p, and miR-142-5p expressions were consistently down-regulated in both datasets, among which the changes of miR-146-3p expression were the most significant (Figure 1a-c). Considering miR-146-3p is involved in regulating the proliferation and migration of cells [8,9], we were interested in the function of miR-146b-3p in VSMCs, and supposed that miR-146b-3p might be a crucial regulator in the dysfunction of VSMCs.

(a-c) GSE127016 and GSE2655 were reanalyzed, the results of which suggested that multiple miRNAs, including miR-146b-3p, probably participated in the pathogenesis of AS. Volcano map displayed all detected miRNAs. MiRNAs with significant alterations of expressions (*P* < 0.05) and log2FC<-1 were marked in blue, and miRNAs with significant alterations of expressions (*P* < 0.05) and log2FC>1 were marked in red. Heat map displayed the 6 miRNAs (miR-146b-3p, miR-142-5p, miR-15b-3p, miR-708, miR-342-3p, and miR-488) with differential expression in both datasets.

### 3.2 The expression of miR-146b-3p was down-regulated by PDGF-BB

**Figure 2.**
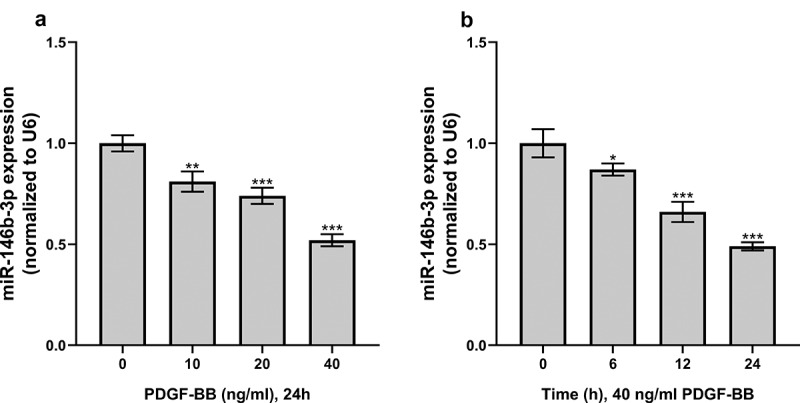
The effect of PDGF treatment on the expression of miR-146b-3p in VSMCs

PDGF-BB can induce the proliferation and phenotypic switch of VSMCs [5]. So, we established a VSMC dysfunction model induced by PDGF-BB to investigate the expression of miR-146b-3p in VSMCs. In this work, after the treatment of VSMCs with different concentrations (0, 10, 20, and 40 ng/ml) of PDGF-BB, it was observed that miR-146b-3p expression was continuously reduced along with the augmentation of PDGF-BB concentration (Figure 2a). In addition, after treating VSMCs with 40 ng/ml PDGF for 0, 6, 12, and 24 h, the expression of miR-146b-3p decreased significantly in a time-dependent manner, and the expression level reached the lowest after 24 h (Figure 2b). The condition that 40 ng/ml PDGF-BB was used to treat VSMC cells for 24 h was hence chosen for follow-up experiments.

(a) The expression of miR-146b-3p was quantified employing qRT-PCR after VSMCs were treated with different concentrations of PDGF for 24 h. (b) The expression of miR-146b-3p was detected by qRT-PCR after VSMCs were treated with 40 ng/ml PDGF for different times.*, **, and *** denote *P* < 0.05, *P* < 0.01, and *P* < 0.001 in comparison with 0 ng/ml group or 0 h group, respectively.

### 3.3 MiR-146b-3p inhibited phenotypic switch and ameliorated proliferation and migration induced by PDGF-BB

**Figure 3.**
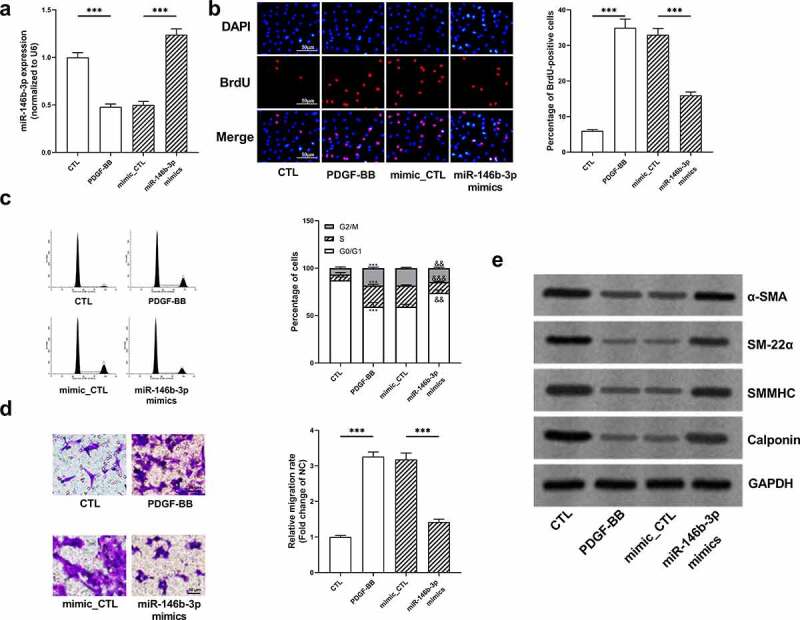
The effect of miR-146b-3p on VSMCs

To investigate the function of miR-146b-3p in VSMCs, miR-146b-3p mimics were transiently transfected into VSMCs, and the transfection efficiency was verified employing qRT-PCR (Figure 3a). BrdU assay manifested that PDGF-BB remarkably facilitated the proliferation of VSMCs, whereas miR-146b-3p overexpression inhibited the proliferation of VSMCs (Figure 3b). Flow cytometry analysis indicated that miR-146b-3p overexpression reversed G2/M cell cycle arrest induced by PDGF-BB (Figure 3c). In Transwell experiment, PDGF-BB markedly increased the migration ability of cells, while miR-146b-3p overexpression significantly impeded the migration of VSMCs (Figure 3d). Besides, Western blot showed that PDGF-BB treatment significantly inhibited the expression of α-SMA, SM22α, SMMHC, and Calponin at protein level, whereas overexpression of miR-146b-3p significantly reversed the regulatory effects of PDGF-BB on these markers of VSMC differentiation (Figure 3e, Supplementary Figure S1a-d). Collectively, overexpression of miR-146b-3p could inhibit PDGF-BB-induced VSMCs from contraction phenotype to synthesis phenotype and prevent excessive proliferation and migration of VSMCs.

(a) The transfection efficiency of miR-146b-3p mimics was detected by qRT-PCR. (b) The proliferation of VSMCs was detected by BrdU assay. (c) The cell cycle distribution of VSMCs was analyzed by flow cytometry analysis. (d) The migration of VSMCs was detected by Transwell experiment. Scale bar = 50 μm.(e) Western blot was employed for detecting the expression of VSMC differentiation markers α-SMA, SM22α, SMMHC, and Calponin.*** denotes *P* < 0.001. In c, *** represents *P* < 0.001 in comparison with control (CTL) group. && and &&& represent *P* < 0.01 and *P* < 0.001 in comparison with mimics control (mimic_CTL) group.

### 3.4 The prediction of the downstream mechanism of miR-146b-3p

**Figure 4.**
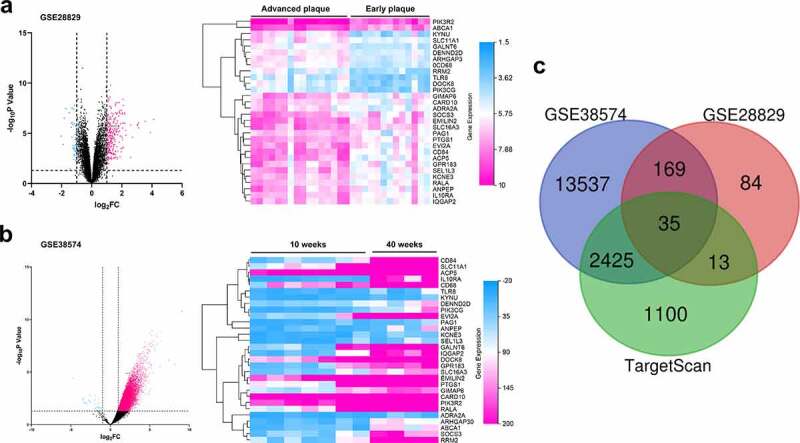
The prediction of pivotal genes in VSMCs which participated in the pathogenesis of AS

To further pinpoint the probable mechanism by which miR-146b-3p regulated the phenotypes of VSMCs, GSE28829 and GSE38574 datasets were utilized to analyze the differential gene expression in AS, and the downstream targets of miR-146b-3p were predicted through TargetScan database. By analyzing the GSE28829, we found that the expression of PIK3CG in advanced AS plaque was markedly higher than that in early AS plaque (Figure 4a). Also, the expression of PIK3CG in mice aortic arch was observed to be remarkably increased after 40 weeks of AS induction in comparison with that in mice after 10 weeks of AS induction in GSE38574 (Figure 4b). Intriguingly, PIK3CG was predicted to be one of the potential downstream targets of miR-146b-3p through analyzing TargetScan database (Figure 4c). PIK3CG, reportedly, can significantly accelerate the proliferation and migration of VSMCs, and promote the transition from contractile VSMCs to synthetic VSMCs [12]. Thereupon, we supposed that miR-146b-3p could probably regulate the phenotypes of VSMCs via repressing PIK3CG expression.

(a-b) GSE38574 and GSE28829 were reanalyzed, the results of which suggested that multiple genes, including PIK3CG, probably participated in the pathogenesis of AS. Volcano map indicated all detected genes. Genes with significant alterations of expression (*P* < 0.05) and log2FC<-1 were marked in blue, while genes with significant alterations of expression (*P* < 0.05) and log2FC>1 were marked in red. Heat map displayed 30 genes with significant alterations (*P* < 0.05) and log2FC>1 in both GSE38574 and GSE28829. (c) PIK3CG was differentially expressed in both GSE38574 and GSE28829, and it was predicted as a target gene of miR-146b-3p by TargetScan database.

### 3.5 PIK3CG was affirmed as the direct target of miR-146b-3p

**Figure 5.**
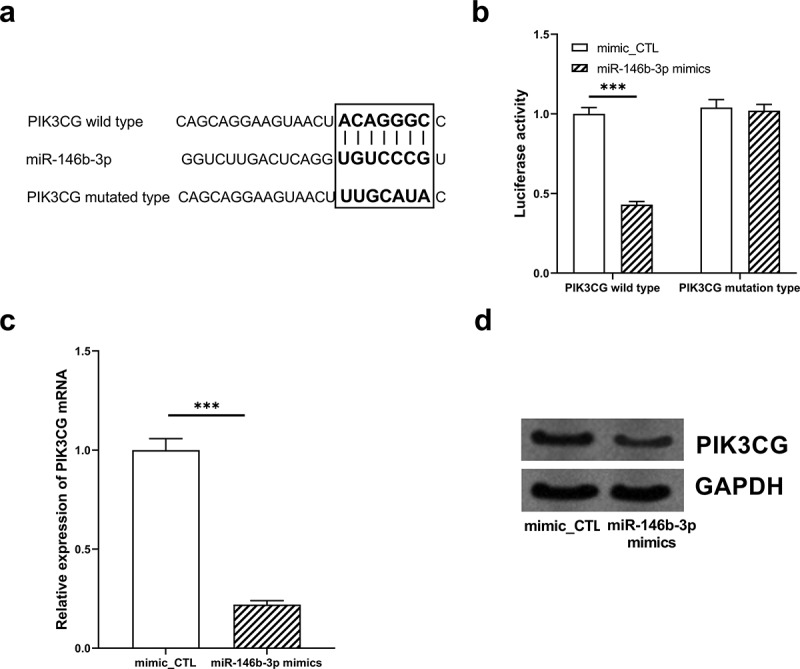
The expression of PIK3CG was inhibited by miR-146b-3p in VSMCs

In order to further verify the targeting relationship between miR-146b-3p and PIK3CG, we constructed PIK3CG 3ʹUTR wild-type (WT) and mutant (MUT) luciferase reporter gene vectors based on the binding sites predicted by the TargetScan database (Figure 5a). Subsequently, dual-luciferase reporter gene assay indicated that the transfection of miR-146b-3p mimics significantly reduced the luciferase activity of WT PIK3CG reporter, whereas no significant effect was observed on that of MUT reporter (Figure 5b). Additionally, following the overexpression of miR-146b-3p, expressions of PIK3CG mRNA and protein were markedly down-regulated in VSMCs (Figure 5c-d, Supplementary Figure 1e). These data authenticated that PIK3CG expression was directly regulated by miR-146b-3p in VSMCs.

(a) The binding sequence between miR-146b-3p and PIK3CG was predicted by TargetScan database. (b) The binding site between miR-146b-3p and PIK3CG was verified by dual-luciferase gene experiment. (c) The expression of PIK3CG mRNA in VSMCs was detected by qRT-PCR after the transfection of miR-146b-3p mimics. (d) The expression of PIK3CG protein in VSMCs was quantified employing Western blot after the transfection of miR-146b-3p mimics. mimic_CTL represents mimic control; *** denotes *P* < 0.001.

### 3.6 The restoration of PIK3CG expression eliminated the effects of miR-146b-3p on VSMCs

**Figure 6.**
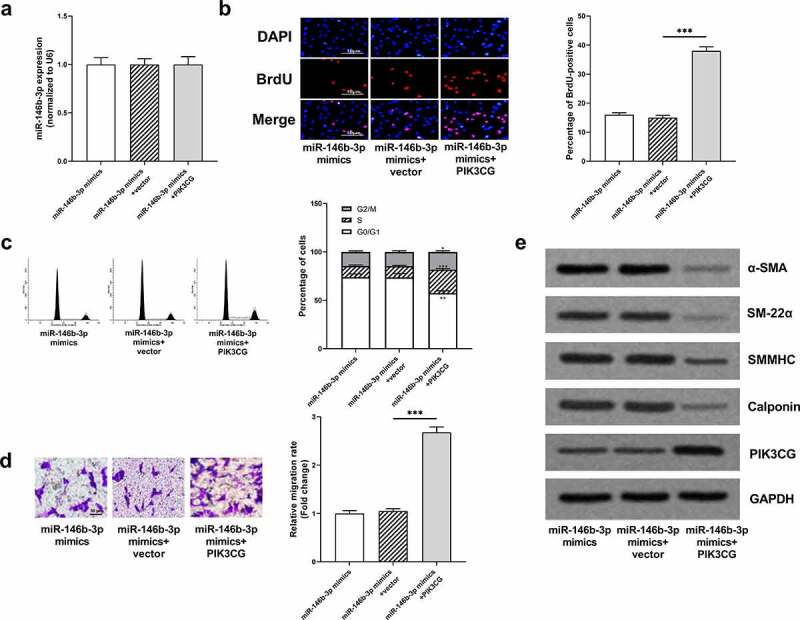
The effects of miR-146b-3p on VSMCs were abrogated by PIK3CG

To further validate our hypothesis, miR-146b-3p mimics and PIK3CG overexpression plasmid were co-transfected into VSMCs, prior to the treatment of VSMCs with PDGF-BB. MiR-146b-3p and PIK3CG expressions were quantified by qRT-PCR and Western blot to verify the success of transfection (Figure 6a, e, Supplementary Figure 1j). The overexpression of miR-146b-3p can significantly inhibit the proliferation, cell cycle progression, and migration of VSMCs induced by PDGF-BB, and the overexpression of PIK3CG reversed the inhibitory effects of miR-146b-3p overexpression and partially rescued the promoting effects of PDGF-BB on VSMCs (Figure 6b-d). Furthermore, expressions of α-SMA, SM22α, SMMHC, and Calponin in miR-146b-3p mimics + PIK3CG group were observably down-regulated compared to these in miR-146b-3p mimics + vector group (Figure 6e, Supplementary Figure 1f-i). Collectively, it was concluded that miR-146b-3p could ameliorate the proliferation and migration and inhibit the phenotypic transformation of VSMCs induced by PDGF-BB via down-regulating PIK3CG expression.

(a) The expression of miR-146b-3p was detected employing qRT-PCR after co-transfection. (b) The proliferation of VSMCs was assessed by BrdU assay. (c) Cell cycle distribution of VSMCs was analyzed employing flow cytometry. (d) Transwell assay was employed for detecting the migration of VSMCs. Scale bar = 50 μm. (e) Western blot was implemented for detecting expressions of α-SMA, SM22α, SMMHC, Calponin, and PIK3CG.*** denotes *P* < 0.001.Vector represents empty vector (pcDNA); PIK3CG represents PIK3CG overexpression plasmids (pcDNA-PIK3CG); *, **, and *** represent *P* < 0.05, *P* < 0.01, and *P* < 0.001, respectively, compared with miR-146b-3p mimics+vector group.

## 4 Discussion

In the pathogenesis of AS, the dedifferentiation of VSMCs from well-differentiated contractile phenotype to synthetic phenotype, and the excessive proliferation and migration of VSMCs, contributes to the initial hyperplasia and formation of AS plaque. Exploring the molecular mechanisms involved in AS development is crucial to develop therapeutic targets for AS. More and more studies substantiate that miRNAs are related to the dysfunction of VSMCs, but the molecular mechanism has not been fully clarified [16]. Some previous studies report the expression characteristics and biological function of miR-146b-3p in cancer biology. For example, in bladder cancer, miR-146b-3p can expedite proliferation and invasion of cancer cells via targeted down-regulation of 15-hydroxyprostaglandin dehydrogenase (HPGD) and neurofibromin 2 (NF2) expressions [17]. Reportedly, expression of miR-146b-3p is remarkably up-regulated in papillary thyroid carcinoma tissues and overexpression of miR-146b-3p significantly potentiates the proliferation, migration, and invasion of BHP10-3 cells [18]. At present, little is known regarding the role of miR-146b-3p in cardiovascular diseases. In our current research, a significant down-regulation of miR-146b-3p expression in a time-dependent and concentration-dependent manner was observed in VSMCs treated with PDGF-BB; overexpression of miR-146-3p up-regulated the expressions of α-SMA, SM22α, SMMHC, and Calponin, implying a protective effect of miR-146b-3p against the dedifferentiation of VSMCs. Intriguingly, it is reported that miR-146b-3p expression is up-regulated after the stimulation of human monocyte THP-1 with TNF-α, which is opposite to the effect of PDGF in down-regulating miR-146b-3p expression in VSMCs; overexpression of miR-146b-3p downregulates the expressions of COX-2 and phosphorylation of p38MAPK in monocytes, which indicates that miR-146b-3p is closely related to inflammatory response [19]. Nevertheless, whether miR-146b-3p can ameliorate inflammatory responses in the microenvironment of AS plaque, deserves further investigation in the following studies.

PI3Kγ is one of the catalytic subunits of PI3K encoded by PIK3CG gene [10,20], and there exist six single nucleotide polymorphisms of PIK3CG, all of which are etiologically associated with the expression level of HDL-cholesterol in plasma, and therefore, the expression of PIK3CG can probably influence the efficiency of HDL clearance, indicating its clinical application in prophylaxis of AS [21]. Additionally, PIK3γ is highly expressed in the aorta of human AS lesions and AS models established by ApoE^−/-^ and LDLR^−/-^ mice; in the animal model of AS established with LDLR^−/-^ mice, PIK3γ specific inhibitor AS605240 can markedly reduce the development of AS [22]. Consistently, in another study, the area of AS lesion in PIK3γ^−/-^ mice, by the comparison with that in PIK3γ^±^ mice, is significantly reduced [23]. Additionally, CD4 + T cells are enriched in AS lesion area, PIK3γ dominates the recruitment of CD4 + T cells, and knockdown of PIK3γ in T cells can ameliorate intimal hyperplasia; knockdown of PIK3γ also represses the expressions of Th1 and Th17 cytokines and alleviates inflammatory responses in VSMCs [24]. PIK3CG is reported to promote the proliferation and migration of VSMCs; mechanistically, PIK3CG activates cAMP responsive element binding protein 1 (CREB-1) and Yes1 associated transcriptional regulator (YAP) [12]. Besides, as the catalytic subunit of PI3K, PIK3CG activates PI3K signaling pathway, and this pathway is verified to promote the proliferation and migration of VSMCs [25]. Also, PI3Kγ-dependent T cell response leads to CXCL10 production in VSMCs, which in turn inhibits endothelial healing [26]. These studies collectively indicate that repressing PI3Kγ expression is a promising strategy for preventing or reversing the pathogenesis of AS. In the present work, PIK3CG was confirmed to be one of the target genes directly regulated by miR-146b-3p, and miR-146b-3p could reduce the expressions of PIK3CG mRNA and protein in VSMCs. Besides, PIK3CG overexpression markedly reversed the regulatory effects of miR-146b-3p overexpression on PDGF-treated VSMCs. Our demonstrations suggest that miR-146b-3p/PIK3CG axis is a novel mechanism involved in VSMCs’ dysfunction and AS pathogenesis.

## Conclusion

Collectively, through *in vitro* experiments, our findings confirm that miR-146b-3p can reduce the proliferation, migration, and phenotypic transformation of PDGF-treated VSMCs via suppressing the expression of PIK3CG. Our study provides a new theoretical basis for regulating VSMCs proliferation and migration, and may contribute to developing new diagnosis and therapy targets for AS in the future. Nonetheless, it is necessary to verify our demonstrations with animal studies in the following work. Furthermore, the relationship between miR-146b-3p/PIK3CG axis and the inflammatory response of VSMCs is needed to be clarified in the future, which will further explain their roles in AS development.

## Supplementary Material

Supplemental Material

## Highlight

1. MiR-146b-3p expression is down-regulated in VSMCs after the treatment of PDGF-BB.

2. MiR-146b-3p inhibits the proliferation and migration of VSMCs and modulates the phenotype switch of VSMCs.

3. The regulatory effects of miR-146b-3p on VSMCs are partly dependent on PIK3CG, and miR-146-3p and PIK3CG are promising targets for the treatment of atherosclerosis.
