# Supplementary figures and images for: MicroRNA-146b-3p regulates the dysfunction of vascular smooth muscle cells via repressing phosphoinositide-3 kinase catalytic subunit gamma
Source: Bioengineered. 2021 Jun 11;12(1):2627–38. doi: 10.1080/21655979.2021.1937904 (PMC8806462; doi:10.1080/21655979.2021.1937904)

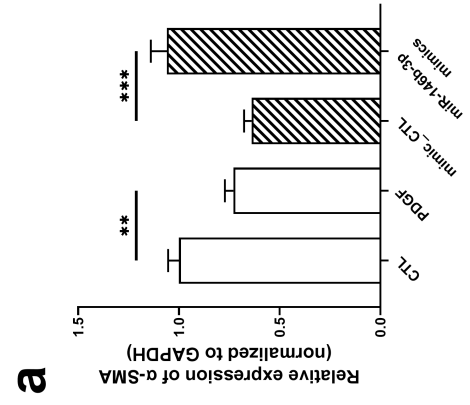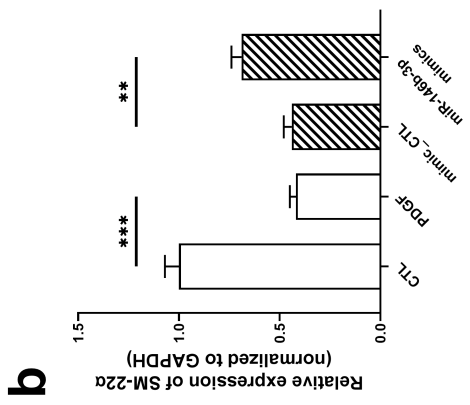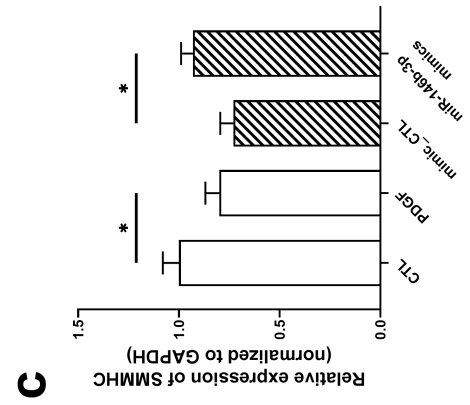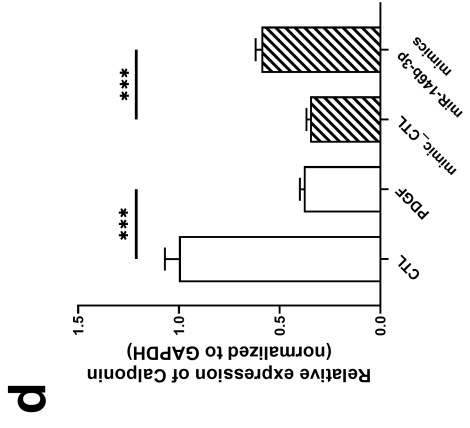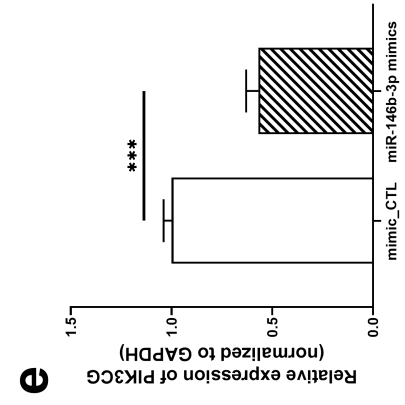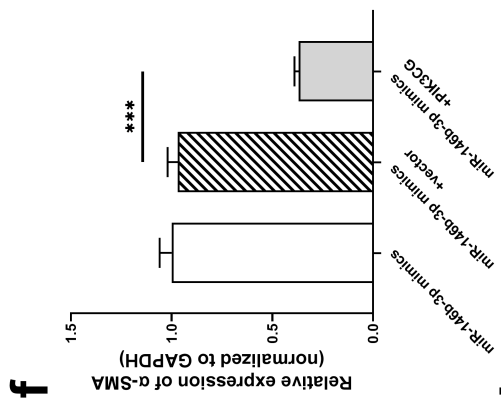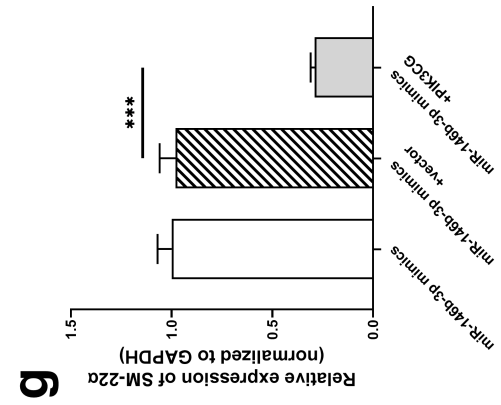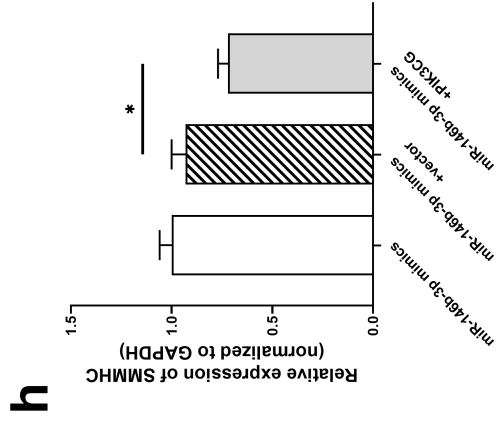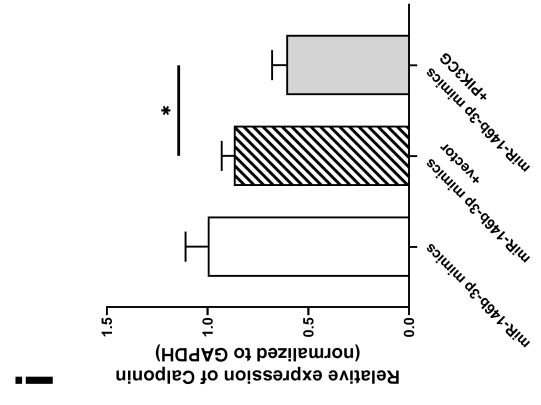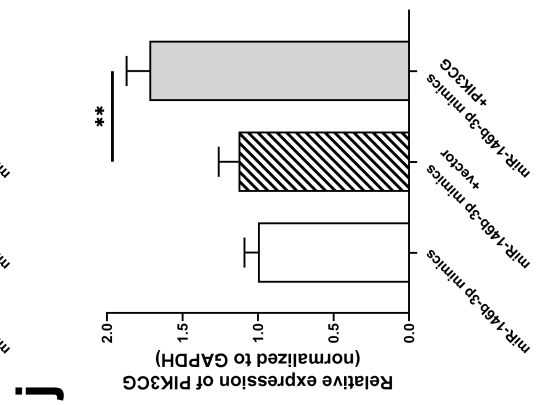

Supplement: Supplemental Material [file KBIE_A_1937904_SM4569.zip › supplementary/downloadFromZipFile.pdf]

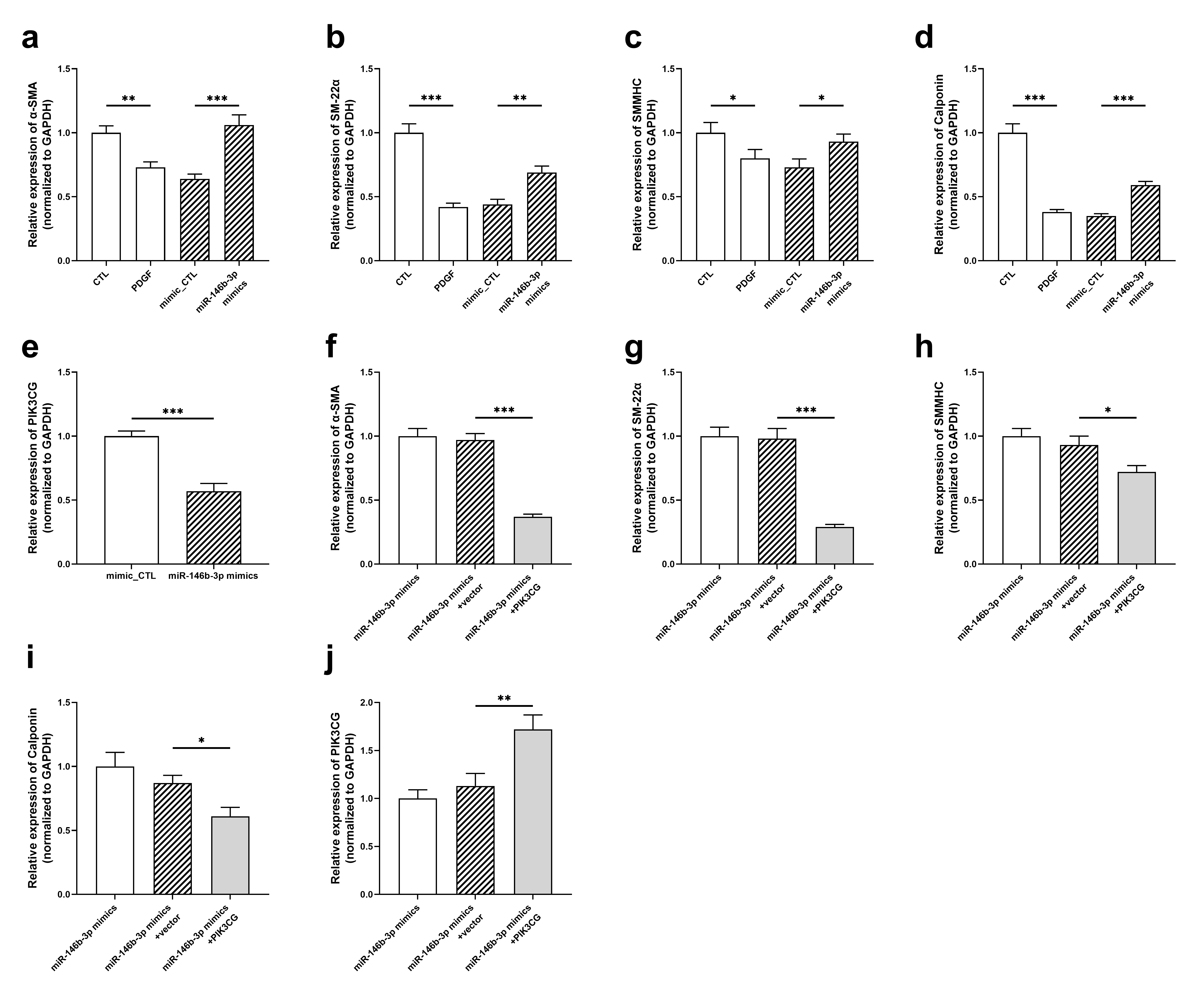

Supplement: Supplemental Material [file KBIE_A_1937904_SM4569.zip › supplementary/Supplementary figure 1_2.tif]
